# Human Campylobacteriosis in Italy: Emergence of Multi-Drug Resistance to Ciprofloxacin, Tetracycline, and Erythromycin

**DOI:** 10.3389/fmicb.2018.01906

**Published:** 2018-08-22

**Authors:** Aurora García-Fernández, Anna M. Dionisi, Sergio Arena, Yaidelys Iglesias-Torrens, Alessandra Carattoli, Ida Luzzi

**Affiliations:** ^1^Department of Infectious Diseases, Istituto Superiore di Sanità, Rome, Italy; ^2^Servei de Microbiologia, Hospital de la Santa Creu i Sant Pau, Barcelona, Spain

**Keywords:** *Campylobacter jejuni*, *Campylobacter coli*, ciprofloxacin, tetracycline, erythromycin, resistance

## Abstract

*Campylobacter* spp. is one of the main cause of bacterial gastroenteritis in the world. The increase of antibiotic resistance in this species is a threat to public health. A *Campylobacter* spp. surveillance study was performed in Italy in the 2013–2016 period by the Enter-Net Italia network. The most prevalent *Campylobacter* species identified causing gastroenteritis was *Campylobacter jejuni* (73.4%) and 45% of all the annual cases of campylobacteriosis were reported in the summer period. High rates of ciprofloxacin and tetracycline resistance in *Campylobacter* spp. have been observed. An increasing percentage of *Campylobacter coli* strains simultaneously resistant to ciprofloxacin, tetracycline and erythromycin has been found. Molecular mechanisms of resistance have been investigated and the role of efflux pumps evaluated. Antibiotic resistance in *Campylobacter* spp. is an increasing serious threat that requires coordinated action to minimize the emergence and spread of antimicrobial resistant strains from animals to humans throughout the food chain.

## Introduction

*Campylobacter* spp. is considered one of the most common cause of bacterial gastroenteritis in the world, and *Campylobacter jejuni* and *Campylobacter coli* are the most common bacterial species isolated from human stool samples (Havelaar et al., 2009; Haagsma et al., 2013; Gibbons et al., 2014; European Food Safety Authority and European Centre for Disease Prevention and Control, 2017). In 2016, 246,307 laboratory-confirmed cases of campylobacteriosis were reported in EU with a notification rate of 66.3 per 100,000 population, 6.1% higher than in 2015 (European Centre for Disease Prevention and Control, European Food Safety Authority, and European Medicines Agency, 2017). *Campylobacter* spp. infections are self-limiting diseases, and treatment with antimicrobials is not recommended. However, antibiotic therapy is indicated for patients with persistent fever, bloody or persistent diarrhea. HIV-positive or immunocompromised individuals normally receive antibiotic treatment if infected by *Campylobacter* spp. (Cha et al., 2016; European Food Safety Authority and European Centre for Disease Prevention and Control, 2017).

Poultry is described as the main reservoir and common source of transmission of campylobacteriosis to humans (Kaakoush et al., 2015). Other risk factors include the consumption of meat, unpasteurized milk and water, contact with animals and international travels, but very rarely human-to-human transmission is reported (Iovine, 2013).

*Campylobacter* spp. exhibits intrinsic resistance to novobiocin, bacitracin, vancomycin, and polymyxins, presumably due to the absence of appropriate targets and/or low affinity binding sites for these drugs (Iovine, 2013). Azithromycin or erythromycin are the first line of choice for antibiotic treatment of campylobacteriosis. Ciprofloxacin and tetracycline are alternatives but not used for treatment of children. In the last decade, ciprofloxacin and tetracycline resistance rapidly increased in *Campylobacter* spp. in humans in Europe, while erythromycin resistance levels remained relatively low (European Food Safety Authority and European Centre for Disease Prevention and Control, 2017). The major mechanism of tetracycline resistance in *Campylobacter* spp. is the binding and protection of ribosomal A site by the protein TetO. Ciprofloxacin resistance in *C. jejuni* and *C. coli* is mainly due to point mutations in the quinolone resistance-determining region (QRDR) of the GyrA protein (Iovine, 2013). Erythromycin resistance in *Campylobacter* spp. has been associated with mutations in the 23S rRNA and in the large loop of the L4 and L22 50S ribosomal proteins (Hao et al., 2013). Multidrug efflux can also contribute to reduce the intracellular concentration of several antibiotics, including tetracycline, ciprofloxacin, and erythromycin (Iovine, 2013).

In Italy, enteric bacterial pathogen surveillance is carried out by a voluntary, passive, laboratory-based, surveillance system named Enter-Net Italia (www.iss.it/site/rmi/enternet) that collects *Escherichia coli, Listeria, Salmonella, Campylobacter, Shigella*, and *Yersinia* records and strains. It is constituted by a network of 27 regional reference laboratories and clinical centers of the national health system. Twenty centers are from Northern (covering 23.8 million people), 4 from Central (8.3 millions) and 3 from Southern (11.5 millions) Italy and they are coordinated by the *Istituto Superiore di Sanità* (ISS). Enter-Net Italia contributes data to the Foodborne and Waterborne (FWD) European surveillance system coordinated by the European Centre for Disease Control (ECDC).

Results of the *Campylobacter* spp. Italian surveillance activities in the period 2013–2016 are herein presented together with results of antimicrobial resistance testing and the investigation of the resistance mechanisms.

## Materials and methods

### Data source

The Enter-Net Italia database was analyzed for age, sex, date of disease onset, and travels for both pediatric and adult patients with a positive *Campylobacter* spp. isolation occurred from January 2013 to December 2016. Patient identity was anonymised. No informed consent was requested because this retrospective study was only focused on the bacteria and did not have impact on the patients.

### Bacterial strains and culture conditions and antimicrobial susceptibility testing

Along the 4 years of surveillance, the National reference center at ISS received 647 *Campylobacter* spp. strains, all accompanied by epidemiological records submitted by strain providers in the Enter-Net Italia database. Strains were grown on Columbia agar plates supplemented with 5% defibrinated horse blood (Oxoid, Hampshire, UK) and Vitox (Oxoid Limited, Hampshire, UK) at 42°C under microaerobic conditions (10% CO2, 5% O2, and 85% N2) for 48 h. When not reported in the record, *Campylobacter* species was determined at ISS by multiplex PCRs for *C. jejuni* and *C. coli* and by simplex PCR for the other species, as previously described (Linton et al., 1996; Denis et al., 1999).

Antimicrobial susceptibility was determined by the disc diffusion method on Mueller–Hinton agar with 5% defibrinated horse blood (Oxoid Limited, Hampshire, UK) and 20 mg/L β-NAD (MH-F) (Oxoid Limited, Hampshire, UK) and microaerobic environment, as recommended by European Committee on Antimicrobial Susceptibility Testing (EUCAST). The following antimicrobials (Becton, Dickinson and Company, NJ, USA) were tested: ciprofloxacin (CIP) (5 μg), tetracycline (TET) (30 μg), erythromycin (ERY) (15 μg) and gentamicin (GEN) (10 μg). *C. jejuni* strain ATCC33560 was used as quality control. Breakpoints were determined following the EUCAST v8.1 guidelines (http://www.eucast.org/clinical_breakpoints/) for CIP, TET and ERY, and the CLSI 2018 guidelines for GEN (CLSI, 2018).

### PAβN efflux pump testing

For 12 selected isolates, disc diffusion antimicrobial susceptibility tests were performed as described above adding in parallel two plates, containing 20 or 40 mg/L efflux pump inhibitor (EPI) PAβN (Sigma–Aldrich, Saint Louis, USA), respectively (Payot et al., 2004). *C. jejuni* ATCC33560 fully susceptible strain was used as control.

### Antibiotic resistance mechanism investigation

A loop of bacteria growth on the plate was resuspended in 1 ml of autoclaved water and chromosomal DNA was extracted and purified using the Chelex 100 molecular Biology grade resin (Bio-Rad Laboratories, Milano, Italy), according to manufacturer's instructions. *C. jejuni* and *C. coli gyrA, rplD* (coding for the 50S L4 protein), *rplV* (coding for the 50S L22 protein) genes, and the *cmeR-cmeA* intergenic region (CmeR-BOX) were amplified by PCR using previously described primer pairs (Zirnstein et al., 1999, 2000; Cagliero et al., 2006; Pérez-Boto et al., 2010; Hao et al., 2013). The amplicons were purified using the Wizard PCR Preps DNA purification system (Promega, Madison, WI, USA) and fully sequenced by fluorescent dye-labeled deoxyribonucletide method with an ABI 3730 automatic DNA sequencer (Perkin-Elmer, Foster City, CA). Multiple sequence alignments of nucleotide and/or amino acid sequences were performed by DNAMAN v.5.2.10 software. The sequences were also analyzed using BLASTN and BLASTP software at the NCBI GenBank sequence database (https://www.ncbi.nlm.nih.gov) and compared *in silico* to those of *C. jejuni* NCTC11168 strain (GenBank accession n. AL111168), considered as the wild type reference sequences.

The V region of *23S rRNA* gene (*rrnB* operon) was amplified by PCR, the amplicons, purified using the ISOLATE II PCR and Gel Kit (Bioline, London, UK) were digested with BsaI (5U) and BceAI (1U) (New England Biolabs, Beverly, UK) restriction enzymes, respectively, as previously described (Vacher et al., 2003). The fragments were separated by electrophoresis on a 2% agarose gel, with ethidium bromide, and visualized under UV light. The results were interpreted as follows: Digestion by BsaI of the 316-bp amplicon containing the A2075G mutation led to two subproducts of 201 and 115 bp in contrast to the uncut wild type and A2074C amplicons. The BceAI digestion, produced three fragments of 41, 24, and 251 bp on the wild type and on the A2075G amplified sequences (Vacher et al., 2003).

All ERY and TET resistant strains were screened by PCR for the presence of *ermB* and *tet(O)* genes, respectively (Pratt and Korolik, 2005; Zhou et al., 2016). The *cmeR–cmeA* intergenic region and the conserved CmeR-Box (5′-TGTAATAAAT [or A] ATTACA-3′) were analyzed in 72 strains with the aim to identify mutations potentially associated with ERY resistance, as previously described (Cagliero et al., 2007; Pérez-Boto et al., 2010; Grinnage-Pulley and Zhang, 2015; Zhang et al., 2017)

### Statistical analysis

Statistical analysis was performed using GraphPad Software (GraphPad Software,La Jolla, USA). Fisher's exact test was used to compare differences in the ratios of infection between patient gender and in the ratios of resistance between the two main *Campylobacter* species. The overall rates of resistance of the isolates and their 95% confidence intervals (95% CIs) were calculated. *P* < 0.05 were considered as significantly different.

## Results

### Surveillance results

From January 2013 to December 2016, 4672 records of *Campylobacter* spp. isolation were included in the Enter-Net Italia database, representing 21% of all data collected in this period of surveillance. In most of these records, *Campylobacter* spp. was isolated from feces (n. 4626, confirmed campylobacteriosis cases) but some notifications reported different sources: 26 strains were from blood, one from urine and 19 from unidentified source. *C. jejuni* was the most frequent species (73.4%), followed by *C. coli* (8%), *C. upsaliensis* (0.4%), *C. lari* (0.1%), *C. fetus* (0.09%), *and C. concisus* (0.04%). In 835/4672 (17.9%) isolates the species was not determined and strains were not sent to ISS for identification.

Among 647 (647/4672, 14%) *Campylobacter* spp. strains received at ISS, 583 (90.1%) were classified as *C. jejuni*, 62 (9.6%) *C. coli*, 1 (0.15%) *C. fetus*, and 1 (0.15%) *C. lari*.

**Figure 1:**
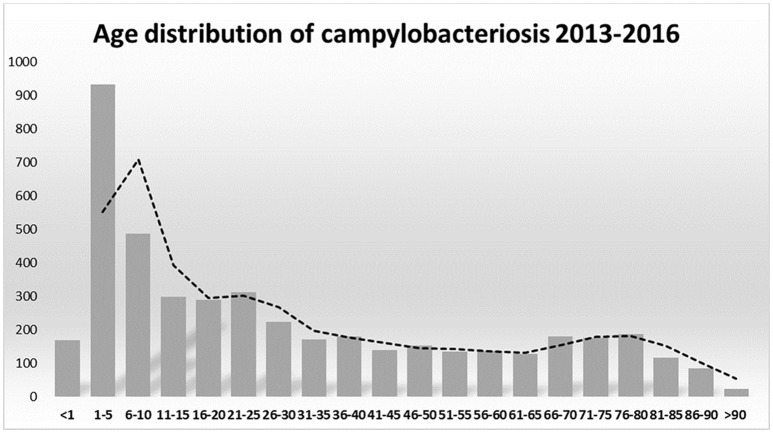
*Campylobacter* spp. notifications by ages 2013–2016.

**Figure 2:**
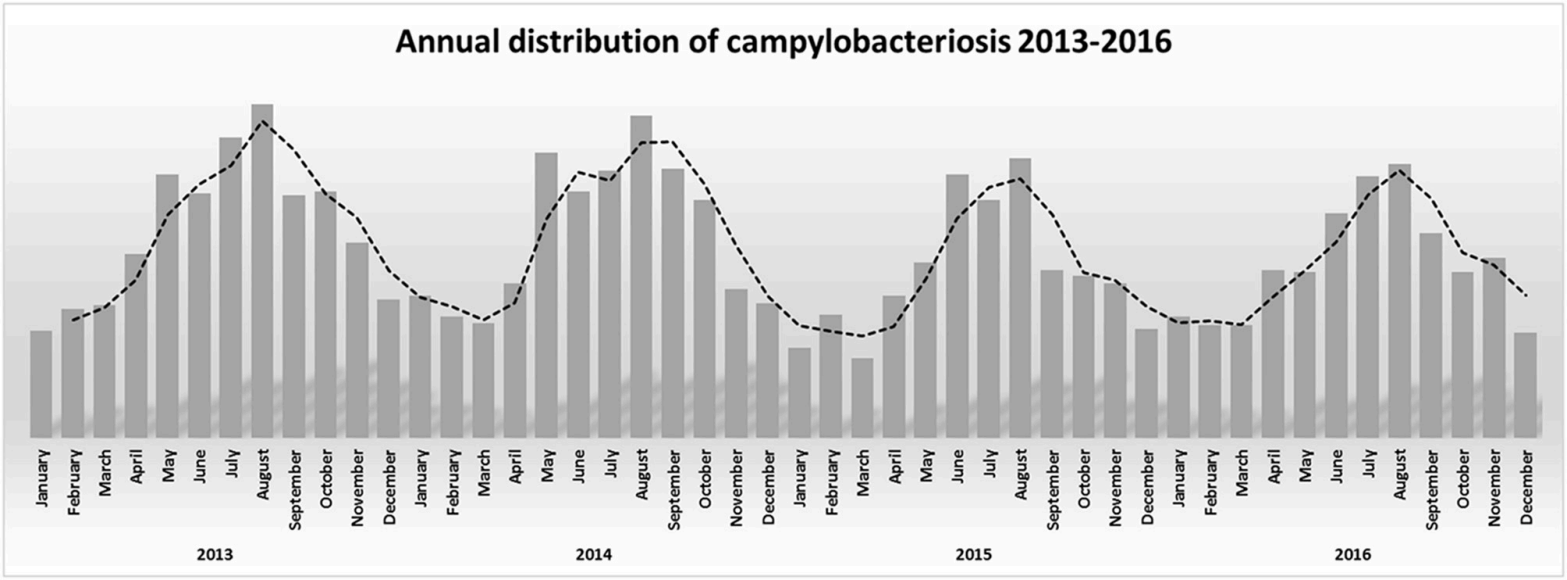
Annual distribution of campylobacteriosis 2013–2016.

The association between campylobacteriosis and gender was statistically significant (*P* < 0.0001), with 2,294 male (49%), 1746 female (37%), and 632 unknown patients reported as positive for *Campylobacter* spp. isolation, respectively. The highest number of *Campylobacter* spp. isolates was obtained from 1 to 5 years old infants with a total of 932 cases (20%) (Figure 1). *Campylobacter* spp. isolation showed a summer peak, with an average of 45% of all the annual cases reported in the June-August trimester (Figure 2). Travels were not significantly associated with a positive *Campylobacter* spp. isolation (217/4672, 5%).

### *Campylobacter* antimicrobial resistance

Antimicrobial susceptibility was determined in 218 of the 647 (34%) *Campylobacter* spp. strains, whose 176 (176/583, 30%) were *C. jejuni*, 41 (41/62, 66%) *C. coli* and 1 *C. fetus*. Strains were selected to represent almost one third of the *C. jejuni* and more than half of the *C. coli* received. *C. coli* was more extensively studied because multi-drug resistance (MDR, defined as resistance to at least three antimicrobial classes) has been described in Europe in this species (European Food Safety Authority and European Centre for DiseasePrevention and Control, 2018). Results showed that 46 (21%) strains (38 *C. jejuni*, 7 *C. coli*, 1 *C. fetus*) were susceptible to all antibiotics tested, and all strains but two *C. jejuni* (0,91%) were susceptible to gentamicin. Differently, 76% of the *C. jejuni* and 70% of the *C. coli* strains were CIP-resistant (CIP-R). Many strains were TET-resistant (TET-R, 64%) and less ERY-resistant (ERY-R, 12%). TET-R and ERY-R strains were more frequently *C. coli* (76% TET-R, 32% ERY-R) than *C. jejuni* (61% TET-R, 7% ERY-R), with *P* < 0.0869 for TET-R and *P* < 0.00001 for ERY-R significant values, respectively.

Among 176 *C. jejuni* isolates, 49.4% were resistant to two, 5.7% to three and 0.6% to four antimicrobial agents, respectively. Of the 41 *C. coli* isolates, 15 (36.6%) strains were resistant to two antimicrobial agents and 15 (36.6%) to three drugs, respectively. The most common resistance patterns were CIP-TET for *C. jejuni* and CIP-TET-ERY for *C. coli*.

### Antimicrobial resistance mechanisms

Seventy-five strains were screened for antimicrobial resistance mechanisms that conferred resistance to CIP, TET and ERY, respectively. Fifty-two *C. jejuni* (5 susceptible, 10 resistant to CIP, 32 to CIP-TET, 3 to CIP-TET-ERY, 1 to TET and 1 to CIP-TET-GEN) and 23 *C. coli* (2 resistant to CIP, 12 to CIP-TET, 1 to CIP-ERY and 8 to CIP-TET-ERY) were tested. All ciprofloxacin-susceptible (CIP-S) strains had wild type GyrA protein sequence, as expected. Among CIP-R strains, the single point mutation Thr86Ile was detected in all but 8 strains. Among them, one showed the Thr86Ala mutation and 3 showed two mutations, Thr86Ile+Asp90Asn, while 4 CIP-R resistant strains did not show amino acid changes in the GyrA deduced protein sequence.

All TET-R strains showed the acquisition of the *tet(O)* gene.

All ERY-R strains were negative for the presence of the *ermB* gene (Table 1). The conserved CmeR-Box (5′-TGTAATAAAT [or A] ATTACA-3′) analysis showed that 20 out of 72 (28%) strains, presented one or more substitutions, insertions and/or deletions respect to the reference sequence of the wild type *C. jejuni* NCTC11168 strain (Table 2). However, most of these mutations were not associated to ERY resistance, being also identified in several ERY-S strains (Table 2). In one ERY-R strain the deletion of the 8th to 11th nt of the 16 bp CmeR-BOX was observed, but its implication in ERY resistance has not yet been proven. More informative results about potential ERY resistance mechanisms were obtained by BsaI and BceAI restriction of the 316-bp PCR amplicon of the 23S rDNA. This analysis demonstrated the presence of the A2075G mutation in 9 ERY-R strains (Vacher et al., 2003).

**Table 1:** Characteristics of antimicrobial resistance mechanisms in 12 the erythromycin-resistant and 3 erythromycin-susceptible *Campylobacter* spp. strains isolated between 2013 and 2016.

**Strain**	**Year**	**Species**	**Resistance**	**E-EPI**	**GyrA**	**Tet(O)**	***23S rRNA***	**L4**	**L22**
91/1/13	2013	*jejuni*	Suscept	nd	wt	nd	wt	wt	wt
19/4/14	2014	*coli*	CIP TET	nd	T86I	POS	wt	V196A	I65V,A74G,S109T,E111A,T114A, A126V, A135V,V137A,K138E^*^
49/1/14	2014	*coli*	CIP TET	nd	T86I	POS	wt	V196A	I65V,A74G,S109T,E111A,T114A, A126V, A135V,V137A,K138E^*^
57/1/13	2013	*coli*	CIP TET ERY	neg	T86I	POS	wt	V196A	I65V,A74G,S109T,E111A,T114A, A126V, A135V,V137A,K138E^*^
74/1/15	2015	*coli*	CIP TET ERY	neg	T86I	POS	wt	V196A	I65V,A74G,A103V,S109A,A135V,V137A,K138E
14/20/15	2014	*coli*	CIP TET ERY	neg	T86I+D90N	POS	wt	V196A	I65V,A74G,A103V,S109A,A135V,V137A,K138E
32/2/15	2015	*coli*	CIP TET ERY	POS	T86I+D90N	POS	A2075G	V196A	I65V,A74G,S109T,E111A,T114A, A126V, A135V,V137A,K138E^*^
7/7/16	2015	*coli*	CIP TET ERY	neg	T86I	POS	A2075G	V196A	I65V,A74G,S109T,E111A,T114A, A126V, A135V,V137A,K138E^*^
42/2/15	2015	*coli*	CIP TET ERY	neg	T86I	POS	A2075G	V196A	I65V,A74G,S109T,E111A,T114A, A126V, A135V,V137A,K138E^*^
55/3/15	2015	*coli*	CIP TET ERY	neg	T86I	POS	A2075G	V196A	I65V,A74G,S109T,E111A,T114A, A126V, A135V,V137A,K138E^*^
3/14/14	2013	*coli*	CIP TET ERY	neg	T86I	POS	A2075G	V196A	I65V,A74G,S109T,E111A,T114A, A126V, A135V,V137A,K138E^*^
57/5/14	2014	*coli*	CIP TET ERY	neg	T86I	POS	A2075G	V121A,V176I,T177S,V184I,M192I,V196A	I65V,A74G,A103V,S109T,E111A, T114A,A126V, A135V,V137A,K138E^*^
6/17/16	2015	*jejuni*	CIP TET ERY	POS	T86I	POS	A2075G	V121A,M192I,V196A	wt
67/1/16	2016	*jejuni*	CIP TET ERY	POS	T86I	POS	A2075G	V121A,M192I,V196A	wt
89/6/13	2013	*jejuni*	CIP TET ERY	neg	T86I	POS	A2075G	V196A	wt

*CIP, ciprofloxacin; TET, tetracycline; ERY, erythromycin; Suscept, Susceptible strain; E-EPI, Erythromycin-Efflux Pump Inhibition test; nd, not determined; POS, positive; neg, negative; wt, wild type (GyrA like ATCC 33560 and 23S rRNA/L4/L22 like NCTC 11168)*.

* *Deletion from 120 to 124 nt (TTTKA) and 130 nt (T)*.

**Table 2:** Partial multiple alignment of the 16 nucleotide intergenic region between *cmeR* and *cmeA* identified in erythromycin-susceptible (ERY-S) and resistant (ERY-R) *C. jejuni* and *C. coli* strains.

**cmeR-BOX**	**ERY-R**	**ERY-S**	**References^a^**
TGTAATAAATATTACA^b^	8	19	Pérez-Boto et al., 2010; Zhou et al., 2016; Zhang et al., 2017
TGTAATAAA**a**ATTACA^b^	1	24	Pérez-Boto et al., 2010; Grinnage-Pulley and Zhang, 2015; Zhou et al., 2016; Zhang et al., 2017
**c**GTAATAAATATTACA	1	1	
TGTAATAAA**a**-TTACA	0	5	Zhou et al., 2016
TGTAATAAA**a**ATTAtA	0	1	Cagliero et al., 2007; Pérez-Boto et al., 2010; Zhou et al., 2016
TGTAATAAATAT**c**ACA	0	2	
TGTAATAAATATT**g**CA	1	3	Grinnage-Pulley and Zhang, 2015; Zhou et al., 2016; Zhang et al., 2017
TGTAATA**----**TTACA	1	0	
TGTAATAAATATT---	0	1	Grinnage-Pulley and Zhang, 2015
TGTAATAAATATTAC**tattac**A	0	3	Pérez-Boto et al., 2010
TGTAATA**tgtgtaata**AATATTACA	0	1	

*Polymorphisms are shown as lower case. Nucleotide deletions are shown as dash. The IR regions are underlined*.

a *References of previously reported mutations*.

b Sequences previously described as wild type or “conserved sequences.”

In 12 ERY-R (9 *C. coli* and 3 *C. jejuni*) and 3 ERY-S strains (2 *C. coli* and 1 *C. jejuni*), the *rplD* and *rplV* genes coding for L4 and L22 50S ribosomal proteins, respectively, were studied. One *C. jejuni*, susceptible to all antimicrobials tested, presented wild type L4 and L22 proteins. The L4 protein showed the V196A mutation in 2 ERY-S and 9 ERY-R strains, respectively. Three ERY-R strains presented additional mutations in the L4 protein, possibly associated with the resistant phenotype (Table 1). Three ERY-R strains exhibited a wild type L22 protein. The other 11 strains showed different mutations in L22, any of them certainly associated with the ERY-R phenotype (Table 1).

### PAβN efflux pump study

The EPI PAβN used at 40 mg/L concentration reduced resistance levels to ERY and TET in some strains, despite the drug did not restore full susceptibility in any of them. The inhibitory effect was dose dependent, being 20 mg/L PAβN less efficacious than 40 mg/L. EPI effect increased the diameter of the inhibition zone for ERY of 9/10 mm in 3 strains (6/17/16, 32/2/15, 67/1/16) and 4 mm in 2 strains (74/1/15, 7/7/16). For TET, an increment of 4/5 mm of the diameter of the inhibition zone was observed in 2 strains (57/1/13, 14/20/15). EPI did not affect ciprofloxacin resistance and bacterial growth at the two concentration tested.

## Discussion

Despite 18% of the records notified in the Enter-Net Italia database did not contain information on species, and strains were not sent to ISS for identification, the study showed a clear prevalence of *C. jejuni* as the main species responsible for campylobacteriosis, followed by *C. coli*. Nevertheless other minor species were reported in the database, but their prevalence can be underestimated because of the lack of identification of numerous strains.

In developing countries, *Campylobacter* spp. infections are more frequent in children under 2 years of age, sometimes resulting in fatal outcomes of complicated infections. In industrialized countries there is a bimodal age distribution for campylobacteriosis, the largest peak at 5 years of age and the second peak at 20–29 years of age (Evans and Brachman, 1998; Butzler, 2004). In Italy *Campylobacter* spp. infection occurred in all age groups, but 1–5 years old children were the most susceptible. A weak increase of campylobacteriosis was also present in young adults and in over 65 years old people (Figure 1). Our epidemiological records reported higher morbidity in males than in females. Other studies also showed a similar gender difference (Butzler, 2004; Schielke et al., 2014; Walter et al., 2018). Seasonality was also observed for campylobacteriosis in Italy, the incidence strongly increased in the summer period, being an independent variable by the year of notification and age groups (Figure 2). Other zoonotic enteric diseases of bacterial origin, e.g., salmonellosis, are also characterized by seasonality, reflecting differences in the risk for exposure to infectious agents during the summer period (Lal et al., 2012; European Food Safety Authority and European Centre for Disease Prevention and Control, 2017).

Currently, macrolides are the drugs of choice for severe campylobacteriosis treatment. Fluoroquinolones are also recommended as the first-line therapy for empirical treatment of undiagnosed diarrheal cases, whereas tetracyclines are considered a second-line treatment. Very high to extremely high resistance levels to ciprofloxacin were reported in human campylobacter isolates in Europe in 2016 (European Food Safety Authority and European Centre for Disease Prevention and Control, 2017). Quinolone resistance in *C. jejuni* from humans in Europe was significantly associated to resistance in *C. jejuni* from food-producing animals, and this resistance was significantly associated to the consumption of quinolones in such animals (European Centre for Disease Prevention and Control, European Food Safety Authority, and European Medicines Agency, 2017). For this reason, fluoroquinolones are currently considered as an inappropriate empirical treatment of human *Campylobacter* spp. infections (Collignon et al., 2016; European Food Safety Authority and European Centre for Disease Prevention and Control, 2017). Resistance to ERY in *C. jejuni* in Europe is generally low (1.5%), while it is higher in *C. coli* (14.4%) that can rise to 24.2–54.5% in several countries. Studies performed in Europe showed that the consumption of macrolides in food-producing animals and resistance in *C. coli* from both food-producing animals and humans was statistically-significant. Equally, resistance in *C. jejuni* from humans was strongly associated with the consumption of macrolides in food-producing animals (European Centre for Disease Prevention and Control, European Food Safety Authority, and European Medicines Agency, 2017; European Food Safety Authority and European Centre for Disease Prevention and Control, 2017). In Italy and in other EU countries, gentamicin resistance was extremely low with 0.9% resistant strains (European Food Safety Authority and European Centre for Disease Prevention and Control, 2017). However, our study showed that in Italy most of the strains (>60%) are CIP-R or TET-R, but also a significant percentage is resistant to macrolides (13%). Furthermore, multidrug resistant *Campylobacter* spp. strains are emerging. It is of public health concern that a high percentage of *Campylobacter* spp. was CIP-TET-R (48% of *C. jejuni* and 41% of *C. coli* isolates) and 29% of the Italian human *C. coli* isolates were CIP-TET-ERY-R.

Several studies performed in *Campylobacter* spp. isolates from broilers and turkeys reared at the slaughters in Northern Italy, showed high resistance levels to quinolones, tetracycline and macrolides. The occurrence of resistant campylobacters in food-producing animals has been associated with the use of antibiotics in animal farming and it is currently considered an important threat to human health because of the high risk of transmission through the food chain of these resistant bugs to humans (Smith and Fratamico, 2010; Giacomelli et al., 2014; Manfreda et al., 2016).

Resistance mechanisms in Italian *Campylobacter* spp. isolates were not univocally identified for all the phenotypes observed. TET resistance was clearly associated to the presence of the *tet(O)* gene, as previously described in most of the *Campylobacter* spp. isolated around the world (Iovine, 2013). Differences of TET resistance between *C. jejuni* and *C. coli* were not statistically significant. Fluoroquinolone resistance in our strains was mainly due to the presence of the Thr86Ile GyrA mutation. This mutation is the most prevalent in clinical and also in veterinary isolates (Iovine, 2013; Wieczorek and Osek, 2013). Previously described mutations in the GyrA protein were also found in two strains of our collection (Wang et al., 1993). Interestingly, four CIP-R strains showed a wild type GyrA sequence. This genotype has been also previously reported in other CIP-R strains (Giacomelli et al., 2014; Tang et al., 2017). It has been hypothesized that in these strains, CIP resistance could be mediated by a chromosomally encoded multidrug efflux pump, which reduces the intracellular concentration of fluoroquinolones (Iovine, 2013; Wieczorek and Osek, 2013). However, we did not measure any effect of the efflux pump inhibitor on wild-type GyrA CIP-R strains, leaving undetermined the mechanism of resistance.

The previously described A2075G mutation in the *23S rRNA* gene, was found in nine out of 12 ERY-R strains tested (3 *C. jejuni* and 6 *C. coli*). Other studies have also indicated that this mutation is usually responsible for high-level ERY resistance (Mamelli et al., 2003; Vacher et al., 2003; Payot et al., 2004; Gibreel et al., 2005; Corcoran et al., 2006). However, ERY resistance mechanism remained undefined in most of our isolates. It has been proposed that modifications in the large loop of the L4 protein (amino acids 55 to 77) and in the highly conserved large loop of the L22 protein (aa 78 to 98) can be associated with macrolide resistance in various bacteria (Cagliero et al., 2006; Hao et al., 2013). In the present study, no variations were found in loop regions of the L4 or L22 proteins. However, the V196A modification, which is located in another region than the large loop of L4, was detected in both ERY-S and ERY-R *Campylobacter* spp. strains, confirming previous reports that did not recognize in this mutation any association with ERY resistance, such as other L4 mutations located outside the large loop (Cagliero et al., 2006; Corcoran et al., 2006). The L22 protein showed several amino acid substitutions and a high variable C-terminal region (amino acids 109 to 138), but these modifications were found in both ERY-R and ERY-S strains and were not associated to macrolide resistance (Cagliero et al., 2006; Pérez-Boto et al., 2010; Zhou et al., 2016). As previously demonstrated, the efflux pump inhibitor PAβN at 20 mg/L had no effect on ERY resistance (Mamelli et al., 2003; Gibreel et al., 2005; Corcoran et al., 2006), whereas a higher concentration of the inhibitor (40 mg/L) led to two- to four-fold decrease in ERY resistance level (Payot et al., 2004). In two ERY-R strains of our collection the pump efflux inhibitor PAβN at 40 mg/L did not restore ERY susceptibility but induced a significant decrement of ERY inhibition halos of the antibiograms, leading to an intermediate inhibition zone. These strains also presented the 23S rDNA mutation A2075G, suggesting that this mutation may contribute to ERY resistance in those strains, together with activation of the efflux pumps. High variability in the CmeR-Box region was found, identifying 11 different sequence variants in our strains. Among them, some were previously described as significantly associated with macrolides resistance (Cagliero et al., 2007; Pérez-Boto et al., 2010; Grinnage-Pulley and Zhang, 2015; Zhou et al., 2016; Zhang et al., 2017). In our strains these modifications were not related to ERY resistance, being also identified in ERY-S strains, and some ERY-R strains showed no mutations in this locus. To our knowledge four of the CmeR-Box variants identified in our study were not previously described. One of them consisted of a deletion of four nucleotides, from nt8 to nt11, into the CmeR binding region, and was detected in one ERY-R strain (89/6/13). This deletion could be associated with deregulation of the *cmeABC* operon expression causing ERY resistance (Table 2). However, this and the other ERY-R strains were negative when they were tested for PAβN inhibition, therefore efflux did not seem relevant for conferring resistance in these strains.

In conclusion, the Enter-Net surveillance improves knowledge and provides novel information on campylobacteriosis in Italy. The present study showed that antibiotic resistance in *Campylobacter* spp. in humans is a worrisome and an underreported problem. The high rates of CIP resistance in *Campylobacter* spp. and the increasing levels of ERY resistance in *C. coli* have converted *Campylobacter* spp. in one of the species included in the priority list of antibiotic-resistant bacteria, created by WHO, to research and develop new and effective drugs (Tacconelli et al., 2018). Prudent use of antimicrobials in food-producing animals, particularly reducing those critically important for human treatment, is important for food safety and public health. Further surveillance and monitoring studies to control the prevalence and antimicrobial resistance in *Campylobacter* spp. must be performed. The application of innovative approaches, such as genomics and proteomics, will offer new insights into the molecular mechanisms responsible of antimicrobial resistance in *Campylobacter* spp.

## Author contributions

Design of the work was developed and overseen by AG-F. Experimental assays were performed by AG-F, AD, SA, and YI-T. AC and IL were responsible for re-drafting the work and revising it critically for important intellectual content. All authors approved the final submitted draft and had opportunity for editing the document.

### Conflict of interest statement

The authors declare that the research was conducted in the absence of any commercial or financial relationships that could be construed as a potential conflict of interest.
